# *KRAS* mutation status in colorectal cancer to predict response to EGFR targeted therapies: the need for a more precise definition

**DOI:** 10.1038/sj.bjc.6604815

**Published:** 2008-12-09

**Authors:** E Rouleau, F Spyratos, B Dieumegard, J M Guinebretière, R Lidereau, I Bièche

**Affiliations:** 1 Oncogenetic Laboratory, INSERM U 735, Centre René Huguenin, Saint Cloud, France; 2 Department of Medicine, Gastroenterology, Centre René Huguenin, Saint Cloud, France; 3 Department of Pathology, Centre René Huguenin, Saint Cloud, France

**Sir,**

The *KRAS* mutations in colorectal cancer (CRC) are associated with clinical resistance to treatment with the epidermal growth factor receptor – targeted monoclonal antibodies. The clinical confirmation of these findings in independent retrospective reports as well as in a phase III trial has led the European Medicines Agency (August 2008) to license panitumumab and cetuximab only for patients with CRC without *KRAS* mutations (http://www.emea.europa.eu/humandocs/Humans/EPAR/erbitux/erbitux.htm and http://www.emea.europa.eu/humandocs/Humans/EPAR/vectibix/vectibix.htm). In the European Public Assessment Report, the natures of the *KRAS* mutations were described as ‘certain hot-spots (mainly codons 12 and 13)’.

The most recent clinical studies report seven somatic mutations in these two codons 12–13 (Amado *et al*, 2008; Lievre *et al*, 2008). They use either direct sequencing (Lievre *et al*, 2008), which addresses few codons surrounding codons 12–13 or focus techniques like Scorpion-ARM (Amado *et al*, 2008), which detects only mutations on codons 12–13 with a high sensitivity.

From March 2008, our genomic platform has tested 70 CRC patients for *KRAS* status. Using a pre-screening approach in codons 12–13 with high-resolution melting curve analysis (Simi *et al*, 2008) followed by sequencing, we found 35% cases with a mutation in codons 12–13, in agreement with the literature. However, we also report two cases presenting a mutation in codon 19 (c.57G>C, p.Leu19Phe), without mutations in codons 12–13. No polymorphism has been reported in this codon (dbSNP – http://www.ncbi.nlm.nih.gov/SNP/). In the two cases, the intensity of the mutated allele in the sequence electropherogram was under 30% of the normal allele. With a heterozygous polymorphism, the intensity between mutated and normal allele should have been similar.

A functional study reported a similar mutation (c.57G>T, p.Leu19Phe) as an activating mutation with proliferative consequences (Akagi *et al*, 2007). In the large Netherlands Cohort Study (*n*=737 CRC), 37% mutations were found in codons 12–13 and 6.6% were found outside codons 12–13, in codons 8, 9, 10, 15, 16, 19, 20 or 25 (Brink *et al*, 2003). A recent report in a Polish population stressed also the finding of mutations in codons 59, 61, 117 in 163 CRC (Wojcik *et al*, 2008). Those mutations cannot be detected with focus techniques. None of those mutations were quoted either in the recent clinical reports assessing the sensitivity to treatment (Amado *et al*, 2008; Lievre *et al*, 2008), or in the Scientific Discussion at the EMEA.

Most clinical studies limited the test to *KRAS* codons 12–13, but additional *KRAS*-activating mutations could be considered in the choice of the treatment. In France, 36 000 new cases of CRC have been estimated each year, leading to potentially 800 patients harbouring false wild-type *KRAS* status and which would not respond to targeted treatments. Activating mutations in codons other than 12–13 raise the question of a precise definition of *KRAS* mutation status in a routine laboratory and clinical practice.
